# Insights Into Mechanisms of Antimicrobial Photodynamic Action Toward Biofilms Using Phenalen-1-One Derivatives as Photosensitizers

**DOI:** 10.3389/fmicb.2020.589364

**Published:** 2020-10-30

**Authors:** Denise Muehler, Christina M. Rupp, Sercan Keceli, Christoph Brochhausen, Heiko Siegmund, Tim Maisch, Karl-Anton Hiller, Wolfgang Buchalla, Fabian Cieplik

**Affiliations:** ^1^Department of Conservative Dentistry and Periodontology, University Hospital Regensburg, Regensburg, Germany; ^2^Institute of Pathology, University Hospital Regensburg, Regensburg, Germany; ^3^Department of Dermatology, University Hospital Regensburg, Regensburg, Germany

**Keywords:** biofilm, antimicrobial photodynamic therapy, SAPYR, chlorhexidine, reactive oxygen species, phenalen-1-one

## Abstract

**Introduction:**

In view of increasing resistance against antibiotics and antiseptics, antimicrobial photodynamic therapy (aPDT) may be a promising approach for use in dentistry. The aim of this study was to investigate the mechanism of action of aPDT with the phenalene-1-one derivatives SAPYR and SA-PN-05 as photosensitizers by evaluating bacterial ability to replicate, membrane integrity, metabolic activity, and formation of reactive oxygen species (ROS) in biofilms of *Actinomyces naeslundii*, *Streptococcus mutans*, and *Escherichia coli*.

**Materials and Methods:**

Single-species biofilms (*A. naeslundii*, *S. mutans*, and *E. coli*) were cultured under aerobic conditions for 48 h followed by treatment with the photosensitizers SAPYR and SA-PN-05 at various concentrations (0, 50, 100, 500 μM) and different incubation periods of 5, 10, 20, and 30 min and subsequent irradiation for 10 min (Waldmann PIB 3000; λ_em_ = 360–600 nm; 50 mW/cm^2^; 30 J/cm^2^). Control samples were treated with dH_2_O and kept in dark for the same periods. Bacterial ability to replicate was evaluated by colony forming unit (CFU) assay. The cytoplasmic membrane integrity was investigated by flow cytometry using SYBR Green and propidium iodide and visualized by scanning and transmission electron microscopy. For SAPYR, metabolic activity and formation of intracellular ROS after irradiation were evaluated via luminescence and fluorometric assays, respectively.

**Results:**

SAPYR showed antimicrobial effects (>3 log_10_ CFU reduction) on *S. mutans* after 5 min and on *A. naeslundii* after 20 min incubation and light activation. For *E. coli*, CFU reduction was >2 log_10_ after 30 min of incubation. SA-PN-05 showed an antimicrobial effect after 5 min for all bacteria. Membrane damage upon aPDT with SAPYR was observed for *E. coli*, but not for *S. mutans* and *A. naeslundii*. Following treatment with SA-PN-05, irradiated samples and dark controls of all three species showed loss of membrane integrity. Luminescence and fluorometric assays showed a reduction in metabolic activity and an increase in formation of intracellular ROS in all three species upon aPDT treatment with SAPYR.

**Conclusion:**

The observed loss in ability to replicate upon aPDT with SAPYR in single-species biofilms may be due to an increase in formation of intracellular ROS upon photodynamic treatment.

## Introduction

The increasing emergence of antimicrobial resistance (AMR) in human and veterinary medicine, food industry, and agriculture and its consequences display a major problem for mankind (Rossolini and Mantengoli, 2008). Currently, over two million people develop infections caused by antibiotic-resistant bacteria every year (CDC, 2019). According to the OECD, around 2.4 million people could die in Europe, North America, and Australia between 2015 and 2050 due to so-called “superbug infections” (OECD, 2018). The WHO even speaks of a possible post-antibiotic era in the 21st century in which common microbial infections could lead to death (WHO, 2014). Therefore, it is crucial to develop new antimicrobial strategies capable of killing resistant bacteria with no risk of inducing resistances themselves.

One alternative to the use of conventional antiseptics and antibiotics is the use of photoantimicrobials as applied in the antimicrobial photodynamic therapy (aPDT). It is based on the treatment of bacteria with a *per se* non-toxic dye, the so-called photosensitizer (PS), and subsequent irradiation with visible light of appropriate wavelength in the presence of molecular oxygen (Wainwright et al., 2017; Cieplik et al., 2018a). The combination of PS, light and molecular oxygen leads to the absorption of a photon by a PS-molecule, achieving an excited singlet state. Then, either charge is transferred to surrounding substrates (type I mechanism), producing superoxide radicals (${\text{O}}_{2}^{-∙}$) that can further react to hydrogen peroxide (H_2_O_2_) and highly reactive free hydroxyl radicals (HO^∙^) or energy is directly transferred to molecular oxygen (type II mechanism) leading to the emergence of singlet oxygen (^1^O_2_) which is considered the most important reactive species in the photodynamic process (Maisch et al., 2007; Cieplik et al., 2018a). The relative amount of type II mechanism is described by the singlet oxygen quantum yield Φ_Δ_ (Maisch et al., 2007; Alves et al., 2014; Baptista et al., 2017; Wainwright et al., 2017). Due to formation of reactive oxygen species (ROS), bacterial structures are destroyed non-selectively by oxidative processes. Therefore, an induction of resistance in bacterial cells is very unlikely after treatment with aPDT (Almeida et al., 2015; Wainwright et al., 2017; Cieplik et al., 2018a). However, for definitely ruling out the possibility of bacterial resistances toward aPDT, it is important to understand the respective mechanism of action of a given PS.

Our group recently found that a newly developed class of PS, phytoalexin-derived phenalen-1-one (PN) derivatives, are effective compounds against both planktonic cultures and biofilms (Cieplik et al., 2013a, 2018b, c; Späth et al., 2014; Tabenski et al., 2016; Muehler et al., 2017). These PS achieve singlet oxygen quantum yields of Φ_Δ_ > 0.9 and therefore react almost exclusively according to type II mechanism (Cieplik et al., 2013a; Späth et al., 2014; Tabenski et al., 2016). This stands in marked contrast to other currently known positively charged PS like the porphyrin-derivative TMPyP, which achieves Φ_Δ_ values around 0.7 (Wilkinson et al., 1993). Since PN derivatives were able to effectively inactivate planktonic bacteria within seconds, it was first hypothesized that the main mechanism of action for aPDT with PN derivatives may be damage of bacterial cytoplasmic membranes (Späth et al., 2014; Tabenski et al., 2016; Muehler et al., 2017). In a previous work, antimicrobial efficacy of aPDT with PN-based PS SAPYR was evaluated on a polymicrobial biofilm of periodontitis-associated bacteria *in vitro* (Cieplik et al., 2018b). In particular, it was investigated whether the mechanism of antimicrobial action of aPDT with SAPYR could be based on cytoplasmic membrane damage. However, flow cytometric analysis combined with propidium iodide (PI) staining revealed no damage of cytoplasmic membranes after aPDT with SAPYR. Furthermore, no uptake of SAPYR by bacterial cells was detected as measured spectrophotometrically (Cieplik et al., 2018b). The exact mechanism of action of aPDT with SAPYR is therefore still not clear. For another phenalen-1-one derivative, SA-PN-05, pronounced antimicrobial efficacy could be observed even without illumination which is probably due to its long alkyl chain (12× C_n_H_2__n+__1_) causing bacterial membrane damage similarly to quaternary ammonium compounds (QACs) such as benzalkonium chloride (BAC) (Muehler et al., 2017).

Therefore, the aim of the present study was to further elucidate the mechanism of action of aPDT using SAPYR and SA-PN-05 as PS. For this purpose, treatment of single-species biofilms with SAPYR was compared to treatment with SA-PN-05 or chlorhexidine digluconate (CHX), which both were employed as positive controls for membrane damage (Cieplik et al., 2018b, c). An antimicrobial assay was used to investigate bacterial ability to replicate and the damage of the cytoplasmic membranes was investigated by flow cytometry. To verify potential membrane damage, scanning electron (SEM), and transmission electron microscopy (TEM) were applied. Furthermore, metabolic activity and the formation of intracellular ROS after treatment were assessed. Revealing the actual mechanism of action of a given antimicrobial approach is of great importance for clinical use and for estimating potential risks of bacterial resistance development toward aPDT by bacteria.

## Materials and Methods

### Chemicals and Light Source

SAPYR {[(1-oxo-1H-phenalen-2-yl)methyl]-pyridinium chloride; λ_abs_ 363-410 nm} and SA-PN-05 {[(1-oxo-1H-phenalen-2-yl)methyl]dodecan-1-aminium chloride; λ_abs_ 360–418 nm} were purchased from TriOptoTec GmbH (Regensburg, Germany), synthesized according to published protocols yielding a purity ≥96% (Cieplik et al., 2013b; Späth et al., 2014; Bauer, 2016; Tabenski et al., 2016; Muehler et al., 2017). PS-solutions were freshly prepared in concentrations of 50, 100, and 500 μM (dissolved in distilled water) and stored in the dark at 4°C for no longer than 2 weeks. For irradiation of SAPYR and SA-PN-05, a gas-discharge lamp (Waldmann PIB 3000; Waldmann Medizintechnik, Villingen-Schwenningen, Germany; λ_em_ 380–600 nm) was used. Irradiance was adjusted to 50 mW/cm^2^ at sample-level, resulting in an energy dose of 30 J/cm^2^ for an irradiation period of 10 min.

**FIGURE 1:**
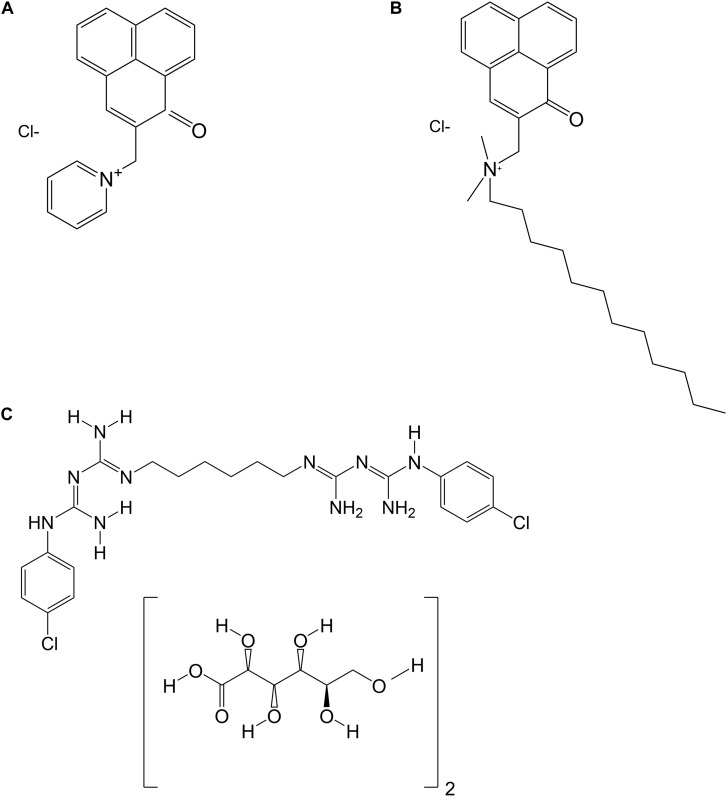
Chemical structures of **(A)** SAPYR; **(B)** SA-PN-05; and **(C)** Chlorhexidine digluconate (CHX).

Chlorhexidine digluconate solution (CHX) was obtained from Sigma-Aldrich (St. Louis, MO, United States) in a concentration of 20% and was dissolved in distilled water to yield the final concentration of 0.2%. Figure 1 shows the chemical structures of SAPYR, SA-PN-05 and CHX.

### Bacterial Culture and Biofilm Formation

Gram-positive *Streptococcus mutans* (DSM 20523; ATCC 25175) and *Actinomyces naeslundii* (DSM 43013; ATCC 12104) as well as Gram-negative *Escherichia coli* (DSM 1103; ATCC 25922) were obtained from the German Collection of Microorganisms and Cell Cultures GmbH (DSMZ; Braunschweig, Germany) and were used as reference strains in this study. *S. mutans* and *A. naeslundii* were grown in Modified Fluid Universal Medium (mFUM) supplemented with 67 mmol/L Sørensen’s buffer (pH 7.2), containing carbohydrate [0.15% (w/v) glucose and 0.15% (w/v) sucrose] and 10% fetal bovine serum (FBS; Gibco Life Technologies, Carlsbad, CA, United States) (Guggenheim et al., 2001; Cieplik et al., 2018c). Tryptic Soy Broth (TSB) was employed as a basal liquid medium for *E. coli* (Villegas et al., 2013). Planktonic cultures (5 mL) were grown aerobically overnight at 37°C.

Afterward, suspensions were harvested by centrifugation (ROTINA 420 R, Hettich Lab Technology, Tuttlingen, Germany) and resuspended in mFUM or TSB yielding an optical density (OD) of 0.1, as measured by means of a spectrophotometer at 600 nm (Ultrospec 3300 pro, Amersham Biosciences, Amersham, United Kingdom). Bacterial suspensions of *S. mutans* and *A. naeslundii* were diluted 1:9 in the biofilm culture medium (BCM) consisting of 50% mFUM, 10% FBS, and 40% whole unstimulated human saliva (saliva) that had been pooled from three volunteers (CMR, SK, and DM; approved by the ethics committee of the University of Regensburg, reference 17-782_1-101) and filter-sterilized (pore size: 0.2 μm; Acrodisc Syringe Filters, Pall, Newquay, United Kingdom), as discussed previously (Cieplik et al., 2018b, c). Bacterial suspensions of *E. coli* were diluted 1:9 in TSB containing 1% glucose.

Single-species Biofilms were formed in 96-well polystyrene culture plates (Corning Costar, Corning, NY, United States). Wells were filled with 200 μL of BCM containing *S. mutans*, *A. naeslundii*, or *E. coli* and incubated under aerobic conditions. After 24 h, medium was carefully removed and 200 μL fresh BCM was added. For all experiments, the total culture period was 48 h.

### Evaluation of Bacterial Ability to Replicate Upon Treatment

After the total biofilm culture period of 48 h, growth medium was carefully discarded from the wells. Then, biofilms were either incubated with 50 μL distilled water or 50 μL SAPYR or SA-PN-05 (50, 100, 500 μM) in the dark for 5, 10, 20, or 30 min and then either illuminated for 10 min or kept in the dark for 10 min. As positive control, biofilms were incubated with CHX (0.2%) for the same period in the dark. Immediately afterward, distilled water, PS, or CHX were carefully removed and the biofilm of each well was brought to suspension with 200 μL phosphate buffered saline (PBS; Biochrom, Berlin, Germany) by frequent pipetting and was transferred to an Eppendorf tube. Complete biofilm removal from the wells was confirmed visually. Then, the Eppendorf tubes were placed in an ultrasonic water-bath chamber (Sonorex Super RK 102 H, Bandelin, Berlin, Germany) obtaining a frequency of 35 kHz for 10 min, and vortexed (REAX top, Heidolph Instruments, Schwabach, Germany) for 5 s to separate aggregated bacteria. Tenfold serial dilutions (10^–2^ to 10^–7^) were prepared in PBS and aliquots (20 μL) were plated on Schaedler blood agar (*S. mutans*, *A. naeslundii*) or Mueller-Hinton agar plates (*E. coli*; both provided by the Institute of Medical Microbiology and Hygiene, University Hospital Regensburg, Germany) according to the method described by Miles et al. (1938). *E. coli* was incubated aerobically for 24 h, while *A. naeslundii* and *S. mutans* were incubated aerobically for 72 h. Subsequently, colony forming units (CFUs) were evaluated (*n* = 6).

### Evaluation of Cytoplasmic Membrane Damage Upon Treatment by Flow Cytometry

For flow cytometry, PI (Sigma-Aldrich) and SYBR Green (SG; Sigma-Aldrich) were used as fluorescent dyes to evaluate integrity of cytoplasmic membranes. Biofilms were prepared, treated and brought to suspension as described above. *A. naeslundii* biofilms were treated for 20 min; *S. mutans* and *E. coli* biofilms were treated for 5 min. After that, samples were centrifuged once at 8000 rpm for 5 min (MiniSpin, Eppendorf, Hamburg, Germany) and resuspended in 0.5 mL PBS. Ten microliters of each sample were mixed with 984 μL PBS and 1 μL SG (100×) and incubated for 15 min in the dark at room temperature. Subsequently, 5 μL PI (5 μg/mL) was added and incubated for another 5 min. After that, samples were immediately processed by a FACSCanto flow cytometer (Becton Dickinson, Franklin Lakes, NJ, United States) equipped with a 488 nm air-cooled solid-state laser with output of 20 mW. PI fluorescence (FL3) was analyzed using a 650 long pass filter, SG fluorescence was analyzed by a 530/30 bandpass filter. Bacterial cells were gated on FSC/SSC dot plots from which FL3/FL1 dot plots were derived. In all cases, 10,000 events were counted. Data acquisition was performed with the FACSDiva 5.0.2 software (Becton Dickinson) and data was analyzed using the FlowJo software, version 10 (FlowJo, LLC; *n* = 6).

### Scanning Electron Microscopy

For scanning electron microscopic visualization, biofilms were prepared on Permanox Chamber Slides (Nunc Lab-Tek Permanox, 4.2 cm^2^/well, Sigma-Aldrich) and treated as described above. *A. naeslundii* biofilms were treated for 20 min; *S. mutans* and *E. coli* biofilms were treated for 5 min. Samples were fixed by adding 2.5% glutaraldehyde buffered with Sørensen’s phosphate buffer (0.1 M; pH 7.4) at room temperature for 2 h. Each slide was washed twice with PBS and three times with distilled water for 15 min each. Then, the fixed samples were additionally dehydrated using 30, 50, 70, 80, 90, 96, and 100% (v/v) graded ethanol, 20 min each. After air-drying overnight in a desiccator, the growth-chambers were removed, and the slides were attached to SEM stubs (∅ 25 mm). For coating, samples were purged with argon and sputtered with platinum for 30 s using a SCD 005 Sputter Coater (Bal-Tec, Balzers, Liechtenstein). Biofilms were examined using a Quanta 400 FEG scanning electron microscope (FEI Company, Hillsboro, OR, United States) in high vacuum mode at 2 kV with 6–7 mm working distance. Tilt and focus were adjusted to ensure optimum viewing. Images were taken from randomly selected fields on the slides.

### Transmission Electron Microscopy

For transmission electron microscopic analyses, biofilms were prepared on small pieces (2 mm^2^) of Thermanox slides (Nunc Thermanox 24 × 30 mm, Science Services, Munich, Germany) and treated as described above. *A. naeslundii* biofilms were treated for 20 min; *S. mutans* and *E. coli* biofilms were treated for 5 min. After treatment, the Thermanox pieces were washed with PBS and then fixed. For *S. mutans* and *A. naeslundii* Karnovsky solution [2.5% glutaraldehyde and 2% paraformaldehyde buffered with cacodylate (0.1 M; pH 7.4)] was used for the fixation, for *E. coli* 4% glutaraldehyde buffered with cacodylate (0.1 M; pH 7.4) was used instead, due to occurring osmotic effects. All samples were then enclosed in 4% low-melting agarose (Invitrogen, Carlsbad, CA, United States). For the embedding process (post-fixation with osmium tetroxide, dehydration, infiltration with EPON – utilizing EM reagents from EMS, Science Services) the LYNX microscopy tissue processor (Reichert-Jung, Wetzlar, Germany) was used. Semi-thin-sections (75 μm), for the selection of relevant areas, and ultra-thin sections (80 nm) were cut using the Reichert Ultracut S Microtome (Leica-Reichert, Wetzlar, Germany). Ultra-thin-sections were then contrasted with aqueous 2%-uranyl-acetate and 2%-lead-citrate solution for 10 min each. The TEM analysis was performed using the LEO 912AB electron-microscope (Zeiss, Oberkochen, Germany).

### Detection of Intracellular ATP Levels Upon Treatment

ATP levels were determined for bacterial biofilms treated with SAPYR and CHX using the BacTiter-Glo Microbial Cell Viability Assay Reagent (Promega, Madison, WI, United States). Biofilms were prepared, treated and brought to suspension as described above. *A. naeslundii* biofilms were treated for 20 min; *S. mutans* and *E. coli* biofilms were treated for 5 min. One hundred microliters of suspensions were mixed with an equal volume of BacTiter-Glo Microbial Cell Viability Assay Reagent in a 96-well opaque plate and incubated for 5 min at room temperature. After incubation, luminescence was measured using a microplate reader (Infinite F200 Microplate reader; Tecan; Männedorf, Switzerland). For calculating ATP concentrations from luminescence intensity values, a standard was prepared according to the manufacturer’s guidelines using adenosine 5-triphosphate disodium salt hydrate (A2383, Sigma-Aldrich). Luminescence was measured for ATP concentrations between 0 and 500 nM for *S. mutans* and between 0 and 1 μM for *A. naeslundii* and *E. coli* (*n* = 4, respectively) and fitted with the end that sigmoidal curves were among best fits (*r*^2^ = 0.97) using Table Curve 2D (Systat Software Inc., San Jose, CA, United States). Additionally, OD at 600 nm (Ultrospec 3300 pro, Amersham Biosciences, Amersham, United Kingdom) of each sample was measured after treatment (*n* = 4). ATP concentrations were then normalized to OD units by dividing ATP concentrations by the OD of the corresponding treated sample.

### Detection of Intracellular ROS Upon Treatment

The intracellular formation of ROS was detected 30 min upon treatment with SAPYR and CHX using 2′7′-dichlorodihydrofluorescin diacetate (H_2_DCF-DA, Themo Fisher Scientific, Waltham, MA, United States). A solution was made by dissolving 2.4 mg of the H_2_DCF-DA powder in 1 mL ethanol (5 mM), which was then diluted to a concentration of 0.5 mM in PBS. The solution was kept in the dark. *A. naeslundii* biofilms were treated for 20 min; *S. mutans* and *E. coli* biofilms were treated for 5 min. Treated biofilms were brought to suspension as described above. Then, 100 μL of each suspension were transferred into a well of a 96-well plate and H_2_DCF-DA was added to a final concentration of 0.25 mM. After incubation for 15 min in the dark, fluorescence of converted 2′,7′-dichlorofluorescein (DCF) was measured at an excitation wavelength of 485 nm and an emission wavelength of 535 nm using a microplate reader (Infinite F200 Microplate reader). From all DCF fluorescence signals the background signal was subtracted (*n* = 4).

### Data Analysis

All CFU results shown as medians, 1st and 3rd quartiles, were calculated using SPSS, v. 25 (SPSS Inc., Chicago, IL, United States) from the values of six independent experiments, each performed in triplicates. Horizontal solid and dashed lines in the graphs represent reductions of 3 and 5 log_10_ steps of CFU, respectively, compared to the untreated control group UC L−. Medians on or below these lines demonstrate an antimicrobial efficacy of 99.9% (3 log_10_) or 99.999% (5 log_10_), at least, which is declared as biologically relevant antimicrobial activity (3 log_10_) or disinfectant effect (5 log_10_) according to guidelines of infection control (Boyce et al., 2002).

ATP levels, DCF intensities and flow cytometric data were calculated as medians, 1st and 3rd quartiles using SPSS. Data were analyzed statistically by applying non-parametric procedures (Mann–Whitney U test) for pairwise comparisons among groups on an α = 0.05 level of significance using SPSS.

## Results

### Evaluation of Bacterial Ability to Replicate Upon Treatment

The bacterial ability to replicate was investigated for biofilms after treatment with aPDT and CHX by a CFU assay. Absolute CFU values (min to max) of untreated biofilms (UC L−) of *A. naeslundii, S. mutans*, and *E. coli* showed growth of 4.9 × 10^7^ to 3.6 × 10^8^; 5.4 × 10^8^ to 1.2 × 10^9^; or 9.5 × 10^6^ to 1.7 × 10^7^, respectively. Table 1 shows the log_10_ reduction rates as compared to untreated controls for SAPYR, SA-PN-05 and CHX for all tested bacteria after irradiation and in the dark. aPDT with SAPYR showed reductions of ≥6.0 log_10_ for *S. mutans* and a concentration−dependent antimicrobial effects for *A. naeslundii* (1.0 to ≥6.0 log_10_). CFU of *E. coli* were reduced by only 2.1 to 2.9 log_10_ after treatment with SAPYR. In all cases, there was no effect from PS or light only (Table 1).

**TABLE 1:** Antimicrobial efficacy of aPDT with SAPYR, SA-PN-05, and of CHX toward *A. naeslundii*, *S. mutans*, and *E. coli* biofilms.

	Incubation period (min)	UC	SAPYR 50 μM		SAPYR 100 μM		SAPYR 500 μM		SA-PN-05 50 μM		SA-PN-05 100 μM		SA-PN-05 500 μM		CHX 0.2%
		L+	L+	L−	L+	L−	L+	L−	L+	L−	L+	L−	L+	L−	L−
*A. naeslundii*	5	0.2	2.5	0.1	3	0.2	5.4	0.4	1.6	4.8	0.8	≥6.0	2.4	≥6.0	4.7
	10	–	1.0	–	1.4	–	2.3	–	1.0	2.4	1.4	≥6.0	2.2	≥6.0	≥6.0
	20	0.2	1.3	–	2.0	–	4.3	–	1.2	1.9	0.9	≥6.0	1.7	≥6.0	≥6.0
	30	0.1	4.4	0.1	4.7	–	≥6.0	0.3	0.2	4.4	–	≥6.0	1.6	≥6.0	≥6.0
*S. mutans*	5	–	≥6.0	0.1	5.3	–	≥6.0	0.71	0.8	1.8	0.9	4.8	4.8	≥6.0	≥6.0
	10	0.3	≥6.0	1.5	≥6.0	1.4	≥6.0	2.0	1.8	2.2	1.5	4.1	4.6	≥6.0	≥6.0
	20	0.3	≥6.0	0.4	≥6.0	0.3	≥6.0	2.3	1.4	1.8	1.9	3.8	3.8	≥6.0	≥6.0
	30	–	5.7	–	5.5	0.2	≥6.0	0.9	1.8	1.5	1.9	3.8	≥6.0	≥6.0	≥6.0
*E. coli*	5	0.7	2.6	0.1	2.4	0.2	2.9	0.4	0.9	0.1	0.6	0.6	1.5	3.7	≥6.0
	10	0.7	2.2	0.1	2.5	0.1	2.5	0.4	1.5	0.3	2.0	0.7	1.8	3.8	≥6.0
	20	0.2	2.1	–	2.3	–	2.6	–	1.4	0.6	1.4	1.2	3.5	≥6.0	≥6.0
	30	0.6	2.2	0.1	2.3	0.2	2.7	–	2.1	0.6	1.6	1.2	3.1	4.3	≥6.0

*Log_10_ reduction of SAPYR, SA-PN-05 and CHX against A. naeslundii, S. mutans, and E. coli for all incubation periods applied as compared to untreated, not irradiated controls (UC L−). L− represents dark groups; L+ represents irradiated groups. – indicates no step reduction of CFU.*

Notably, for SA-PN-05 higher CFU reductions were observed in the dark than upon irradiation. Upon irradiation, aPDT with SA-PN-05 led to reductions of 0 to 2.4 log_10_ against *A. naeslundii*, 0.8 to ≥6.0 log_10_ against *S. mutans* and 0.6 to 3.5 log_10_ against *E. coli*. On the contrary, treatment with SA-PN-05 in the dark led to CFU-reductions of 1.5 to ≥6.0 log_10_ against *A. naeslundii*, 1.9 to ≥6.0 log_10_ against *S. mutans* and 0.1 to ≥6 log_10_ against *E. coli*. For CHX 0.2%, CFU of all three bacteria were reduced by ≥6 log_10_ below the detection limit (Table 1).

**FIGURE 2:**
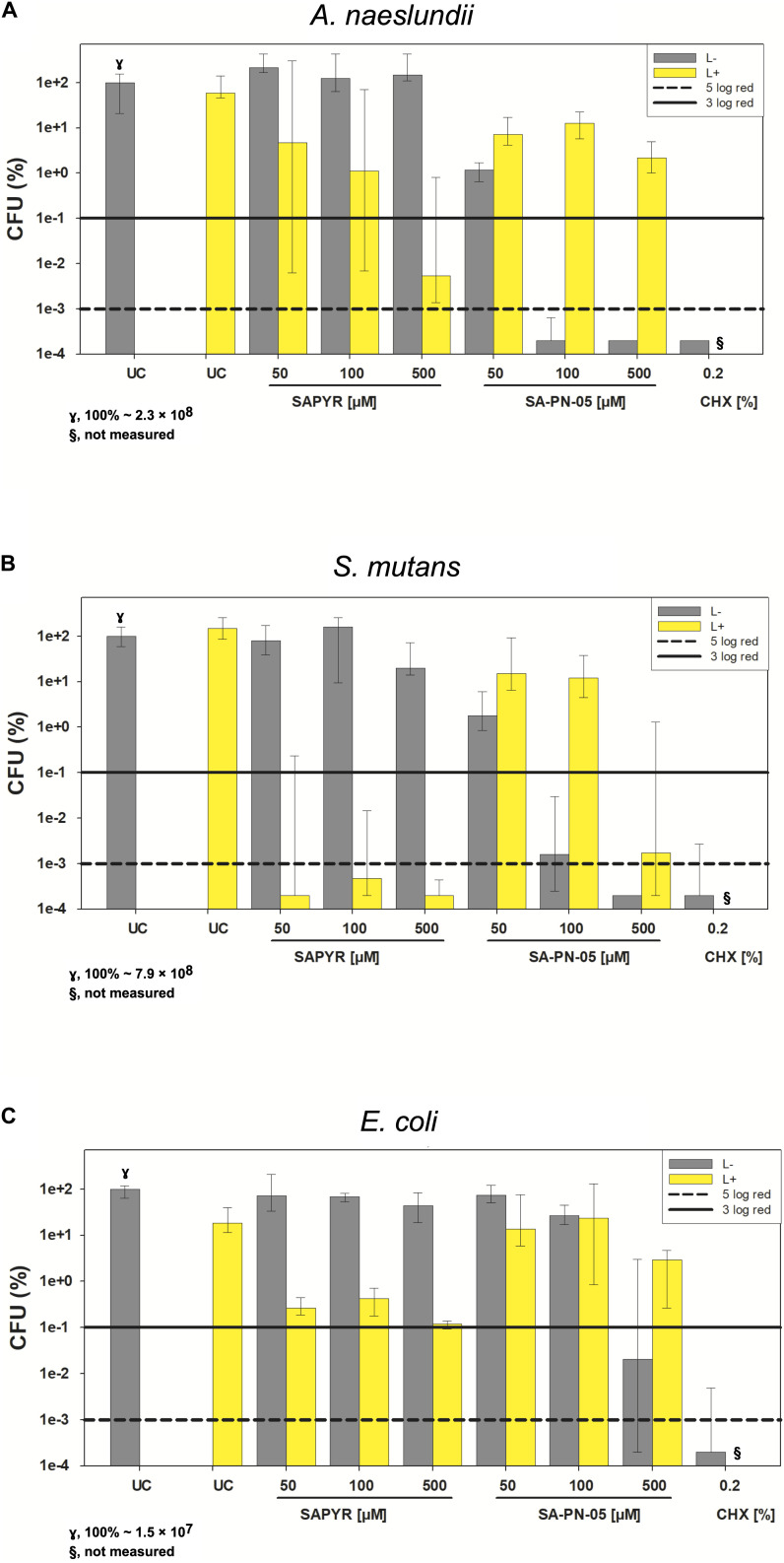
Antimicrobial efficacy of aPDT with SAPYR SA-PN-05 and of CHX toward *A. naeslundii*, *S. mutans*, and *E. coli* biofilms. All results are depicted as medians, 1st and 3rd quartiles on a log_10_-scaled ordinate. L– represents dark groups; L+ represents irradiated groups. Relative CFU data [CFU (%)] with untreated control group (UC L–) set to 100% for all bacteria. Horizontal solid and dashed lines represent CFU-reductions of 3 log_10_ and 5 log_10_, respectively. **(A)** *A. naeslundii* upon treatment with SAPYR or SA-PN-05 for 20 min incubation and 10 min irradiation or with CHX for 30 min incubation (γ, 100% ∼ 2.3 × 10^8^); **(B)** *S. mutans* upon treatment with SAPYR or SA-PN-05 for 5 min incubation and 10 min irradiation or with CHX for 15 min incubation (γ, 100% ∼ 7.9 × 10^8^); **(C)** *E. coli* upon treatment with SAPYR or SA-PN-05 for 5 min incubation and 10 min irradiation or with CHX for 15 min incubation (γ,100% ∼ 1.5 × 10^7^); §, not measured. *n* = 6.

Figure 2 exemplary shows CFU results upon treatment with SAPYR and SA-PN-05 for selected incubation periods [i.e., 20 min for *A. naeslundii* (Figure 2A), and 5 min for *S. mutans* (Figure 2B) and *E. coli* (Figure 2C)]. Relative CFU data [CFU (%)] with untreated controls (groups UC L−) set to 100% are depicted separately for each bacterial strain. All further experiments were carried out using these selected incubation periods.

### Flow Cytometric Evaluation of Cytoplasmic Membrane Damage Upon Treatment

**FIGURE 3:**
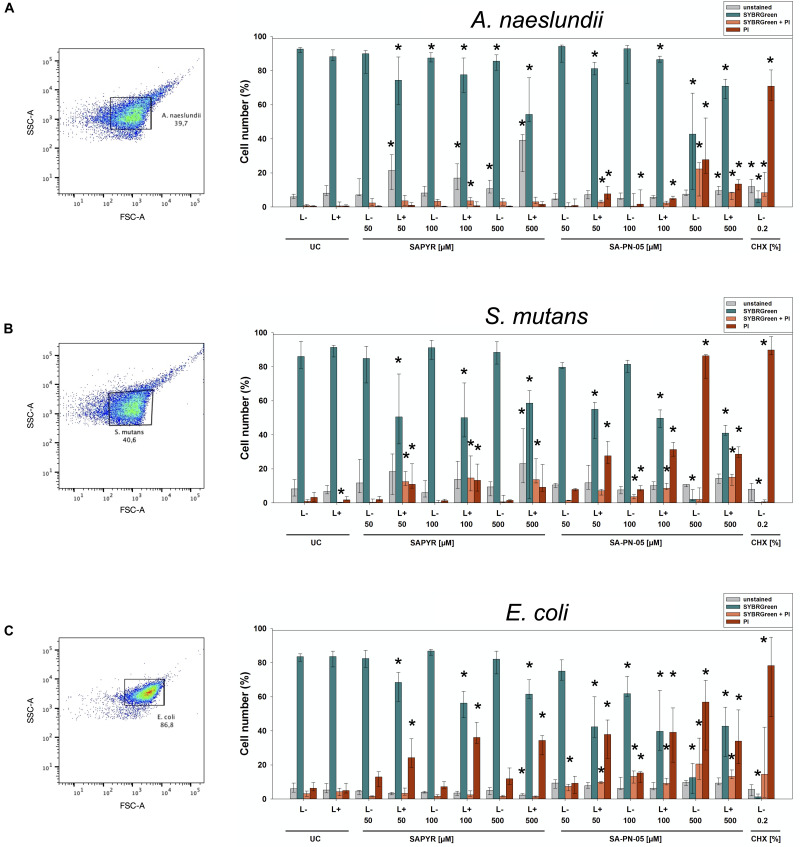
Flow cytometric measurements for membrane integrity upon treatment with aPDT using SAPYR, SA-PN-05, or CHX. Bacterial cell populations gated on dot plot FSC vs. SSC showing the chosen region of interest (ROI) for **(A)** *A. naeslundii*, **(B)** *S. mutans*, and **(C)** *E. coli*. Summarized median percentages, 1st and 3rd quartiles of unstained (gray), SG-stained (turquoise), double stained (orange) as well as PI-stained bacterial cells (red) are shown for all experimental groups **(A–C)**. Asterisk depict statistical difference (*p* ≤ 0.05) among UC L- and treated groups. L- represents dark groups; L+ represents irradiated groups. *n* = 6.

Flow cytometry with PI and SG as fluorescent dyes was employed to evaluate membrane integrity upon treatment with aPDT or CHX. Figure 3 shows the region of interest (ROI) determined in untreated controls for all three bacterial species on dot plots FSC vs. SSC and summarized percentages of unstained, SG-stained, double stained as well as PI-stained bacterial cells for all groups treated with SAPYR, SA-PN-05, and CHX.

For *A. naeslundii*, untreated biofilms (UC L−) showed a proportion of PI-positive cells of 0.5%. Neither treatment with PS or light alone, nor aPDT with SAPYR at all concentrations tested led to a significant increase of PI-positive cells as compared to untreated controls (medians of PI-positive cells: 0.5 to 1.6%). In contrast, there was significant increase in unstained cells after aPDT (6 to 39%). Furthermore, there was a significant increase in PI-positive cells after treatment with SA-PN-05 with and without light to 13.4 and 27.8%, respectively. For *S. mutans*, median of PI-positive cells of UC L− was 3.2%. There was a significant increase in PI-positive cells after aPDT with SAPYR (13.2%). Furthermore, there was a significant increase in unstained cells after aPDT with SAPYR (8.2 to 23%) PI-positive cells after aPDT with SA-PN-05 showed a significant increase to 31.4%; dark controls to 86.4%. UC L− of *E. coli* showed a median of PI-positive cells of 6.4%. Upon treatment with SAPYR, PI-positive cells increased significantly to 36.2%. Upon treatment with SA-PN-05 and light, PI-positive cells increased significantly to 39.2%; while treatment without light led to a percentage of 56.8%. Treatment with CHX led to a median increase of PI-positive cells from 67.9 to 95.2% for all groups.

### Scanning Electron Microscopy

**FIGURE 4:**
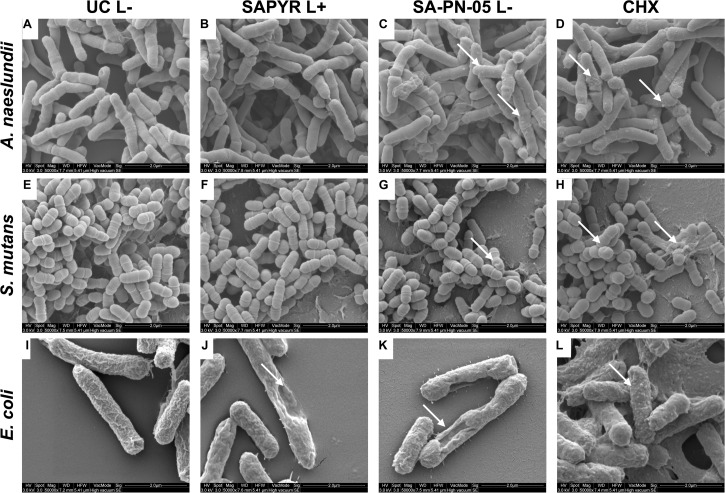
Exemplary visualization of single-species biofilms by means of scanning electron microscopy (SEM). Visualization of single-species biofilms **(A–D)** *A. naeslundii*, **(E–H)** *S. mutans*, and **(I–L)** *E. coli*, either untreated (UC L–) or treated with aPDT using SAPYR (L+; 100 μM); SA-PN-05 (L–; 100 μM); or CHX (L–; 0.2%), at an original magnification of 50,000–fold (scale bar ∼ 2 μm). White arrows depict alterations in membrane structure.

Figure 4 shows exemplary SEM images taken from randomly selected fields of untreated biofilms (group UC L−) and biofilms treated with aPDT with SAPYR (L+; 100 μM) or SA-PN-05 (L−; 100 μM) or CHX (L−; 0.2%). Untreated cells of all three bacterial species showed an intact morphology with a regular cell surface (Figures 4A,E,I). After treatment with aPDT using SAPYR, *A. naeslundii* and *S. mutans* cells showed no alteration in bacterial morphology (Figures 4B,F). In contrast, surfaces of *E. coli* cells looked corrugated, and lysed cells with large holes could be observed (Figure 4J). After treatment with SA-PN-05 and CHX, numerous blister-like structures were found on cell surfaces of *A. naeslundii* (Figures 4C,D). Treated *S. mutans* cells frequently showed holes in their surface (Figures 4G,H), whereas *E. coli* cells showed large holes, blisters and cellular debris (Figure 4K,L).

### Transmission Electron Microscopy

**FIGURE 5:**
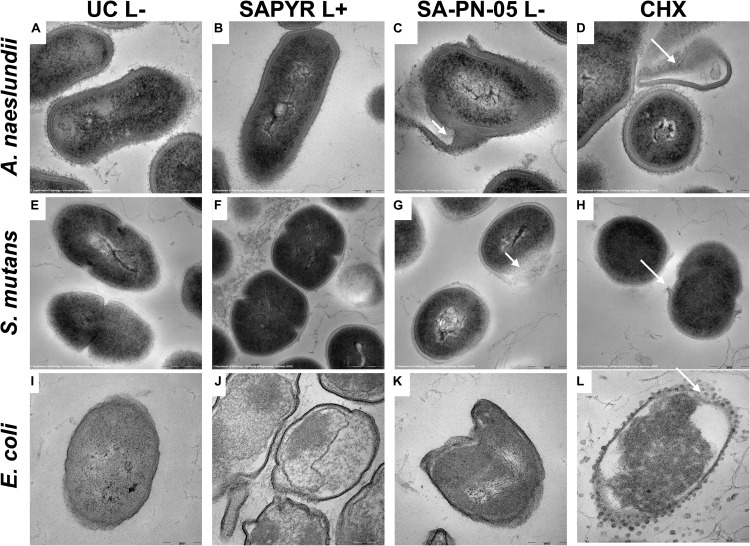
Exemplary visualization of single-species biofilms by means of transmission electron microscopy (TEM). Visualization of single-species biofilms **(A–D)** *A. naeslundii*, **(E–H)** *S. mutans*, and **(I–L)** *E. coli* (48 h), either untreated (UC L–) or treated with aPDT using SAPYR (L+; 100 μM); SA-PN-05 (L–; 100 μM); or CHX (L–; 0.2%), at an original magnification of 100,000–fold (scale bar ∼ 200 nm). White arrows depict alterations in membrane structure.

Figure 5 shows exemplary TEM images taken from randomly selected fields of untreated biofilms (group UC L−) and biofilms treated with aPDT with SAPYR (L+; 100 μM) or SA-PN-05 (L−; 100 μM) or CHX (L−; 0.2%). In all cases, untreated bacteria showed intact cell walls and well-defined membranes (Figures 5A,E,I). No damage of the membrane was detected in *A. naeslundii* and *S. mutans* treated with aPDT and SAPYR (Figures 5B,F). After incubation with SA-PN-05 or CHX without irradiation, both strains exhibited cell wall damage and cytoplasmic content leakage (Figures 5C,D,G,H). *E. coli* cells treated with aPDT and SAPYR showed separation of the cytoplasmic membrane from the cell wall (Figure 5J). Compared to the untreated cells, cell distortion occurred upon treatment with SA-PN-05 in the dark (Figure 5K). After treatment with CHX, blebbing of the outer membrane resulting in formation of vesicles were seen in *E. coli* (Figure 5L).

### Detection of Intracellular ATP Levels Upon Treatment

**FIGURE 6:**
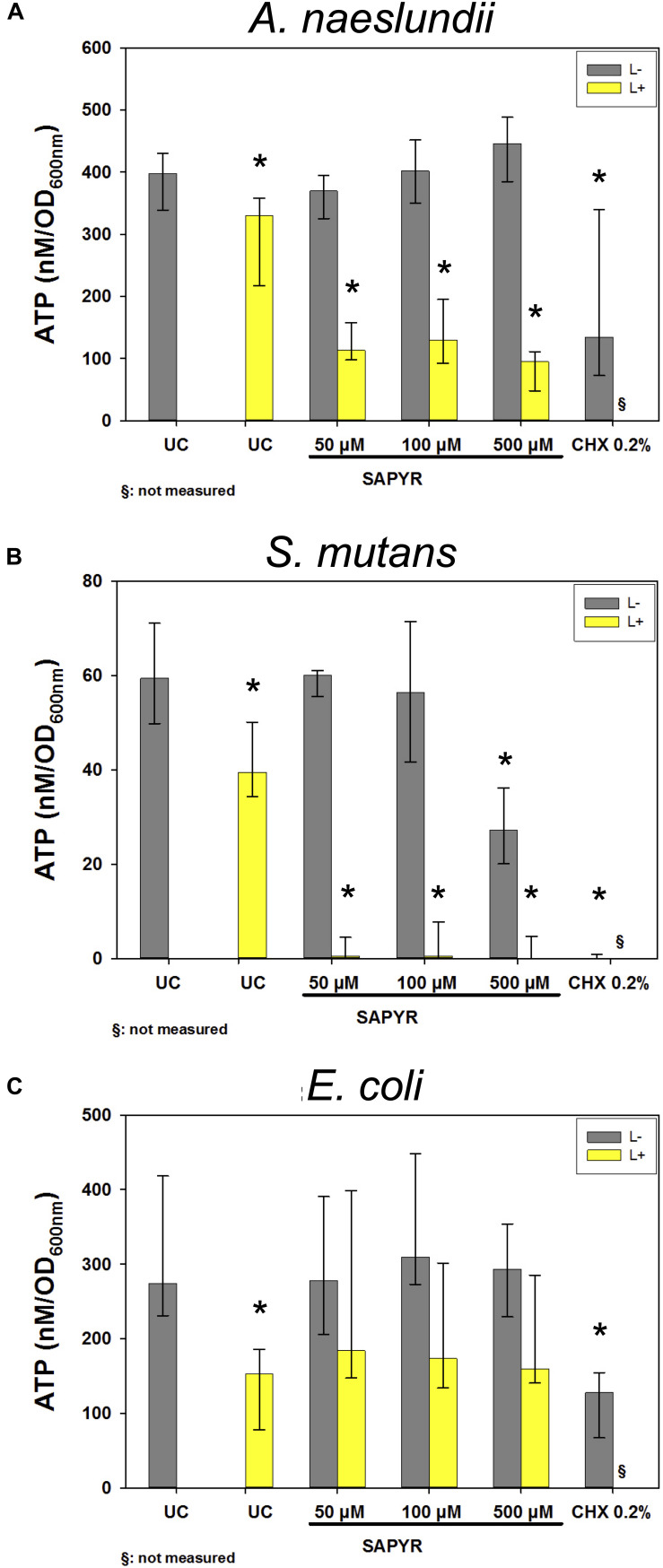
Luminescence measurements for ATP level upon treatment with aPDT using SAPYR or CHX. Depicted are medians, 1st and 3rd quartiles of bacteria treated with SAPYR or CHX. ATP concentration was normalized to corresponding OD measurements of samples after treatment and expressed as ATP (nM/OD_600_) **(A)** *A. naeslundii* after 20 min incubation. **(B)** *S. mutans* after 5 min incubation. **(C)** *E. coli* after 5 min incubation. Yellow bars (L+) represent groups treated with SAPYR irradiated for 10 min. Gray bars (L–) represent dark controls or CHX, respectively. Asterisk depict statistical difference (*p* ≤ 0.05) among UC L– and treated groups. §, not measured. *n* = 4.

Figure 6 shows results of calculated ATP concentrations for *A. naeslundii* (Figure 6A), *S. mutans* (Figure 6B), and *E. coli* (Figure 6C). Dark controls showed no significant changes of ATP concentration except for *S. mutans* with 500 μM SAPYR which led to a significant decrease of 33 nM/OD_600_ compared to untreated controls. Furthermore, exposure to light without SAPYR led to a significant decrease of ATP of 67, 20, and 121 nM/OD_600_ compared to untreated control for *A. naeslundii*, *S. mutans*, and *E. coli*, respectively. Exposure to light with PS led to a decrease of 300, 60, and 121 nM/OD_600_ for *A. naeslundii, S. mutans*, and *E. coli*, respectively. Intracellular ATP levels also decreased significantly for all bacteria upon treatment with CHX.

### Formation of Intracellular Reactive Oxygen Species

**FIGURE 7:**
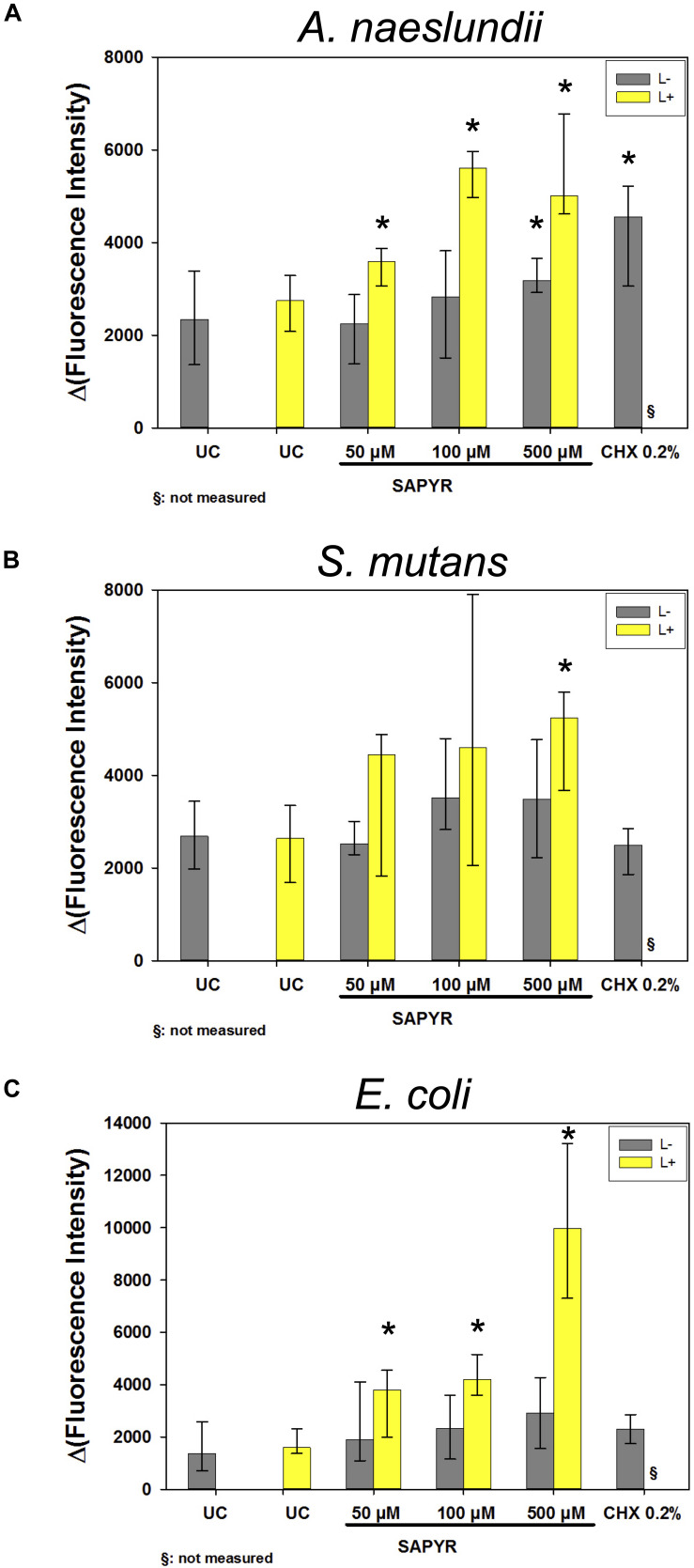
Fluorescence measurements for formation of ROS upon treatment with SAPYR or CHX. Depicted are medians, 1st and 3rd quartiles of Δ(Fluorescence Intensity) which is the measured fluorescence intensity with autofluorescence substracted. **(A)** *A. naeslundii* after 20 min incubation. **(B)** *S. mutans* after 5 min incubation. **(C)** *E. coli* after 5 min incubation. Yellow bars (L+) represent groups treated with aPDT irradiated for 10 min. Gray bars (L–) represent dark controls or CHX, respectively. Asterisk depict statistical difference (*p* ≤ 0.05) among UC L– and treated groups. §, not measured. *n* = 4.

As detected by fluorescence measurements of DCF, the production of intracellular ROS increased in all bacteria 30 min upon treatment with aPDT (Figure 7). Treatment with SAPYR without irradiation led to a concentration-dependent increase in Δ(Fluorescence Intensity) of 833, 833, and 1541 as compared to untreated controls for *A. naeslundii*, *S. mutans*, and *E. coli*, respectively. Treatment with SAPYR and irradiation led to a significant concentration-dependent increase in Δ(Fluorescence Intensity) of 2664, 2551, and 8602 for *A. naeslundii*, *S. mutans*, and *E. coli*, respectively. In all cases, there was no significant effect of treatment with light only. Measurements upon treatment with CHX showed no changes for *S. mutans*, but a significant increase in Δ(Fluorescence Intensity) of 2206 for *A. naeslundii* and an increase of 938 for *E. coli.*

## Discussion

Antimicrobial resistance represents one of the current major problems in medicine and is caused by, among other things, inappropriate use of antimicrobial agents all over the world (Bebell and Muiru, 2014; Roca et al., 2015). Therefore, it is necessary, to not only find alternative antimicrobial approaches but it is even more important to investigate and to understand their mode of action to prevent further spread of resistance. In the present study, we combined measurements of bacterial ability to replicate, membrane integrity, intracellular ATP levels and the formation of intracellular ROS to investigate the mechanism of action of the PN-mediated photodynamic process when applied to biofilms. We focused on the lead-candidate SAPYR which has already shown pronounced antimicrobial efficacy against planktonic cultures and biofilms in previous studies (Cieplik et al., 2013a, 2018b, c; Tabenski et al., 2016; Muehler et al., 2017). As positive control for antimicrobial efficacy, CHX was used as gold-standard antiseptic in a typical concentration applied in dentistry (0.2%) (Cieplik et al., 2019). As positive control for loss of membrane integrity, the PS SA-PN-05 was used which comprises the identical basic chemical PN-structure as SAPYR, but acts by damaging cytoplasmic membranes due to its alkyl chain (Muehler et al., 2017).

For this set of experiments, single-species batch-culture biofilms of the typical oral bacteria *S. mutans* and *A. naeslundii* as well as of *E. coli* were employed as model systems. While biofilms formed in flow cells are mostly used for microscopic examination of biofilm formation under flow conditions, *s*tatic batch-culture biofilm models facilitate high-throughput investigations and thus are commonly used to investigate molecular mechanisms like in the present study (McBain, 2009).

First, a CFU assay was employed for assessing reductions in the bacterial ability to replicate upon the various treatments tested. Treatment with CHX resulted in CFU-reductions by more than 6 log_10_ against all tested bacteria. Similar antimicrobial efficacy was found for aPDT using SAPYR as PS. The photodynamic effect of SA-PN-05 (2.0 to 2.8 log_10_) was clearly inferior as compared to SAPYR (4.4 to 6.1 log_10_) which was also found in a previous study for inactivation of planktonic bacteria (Muehler et al., 2017). In addition, due to its chemical structure, SA-PN-05 showed a dark toxicity in contrast to SAPYR. Its positively charged head group and long alkyl chain (12× C_n_H_2__n+__1_) may enable SA-PN-05 to penetrate the bacterial membrane to cause membrane damage even without illumination (Muehler et al., 2017). On the contrary, SAPYR contains a positively charged methyl-pyridinium group as the respective head group, but no alkyl chain, and thus no diffusion may take place in deeper parts of the lipidic membrane area resulting in no dark toxicity (Muehler et al., 2017). Accordingly, Reddi et al. (2002), showed that dark toxicity increased by adding a longer alkyl chain (up to 14× C_n_H_2__n+__1_) to the porphyrin-based PS T_4_MPyP. For the present study, the higher CFU reduction rates for SA-PN-05 in the dark compared to the irradiated groups may be attributed to a partial break-down of this molecule after irradiation for 10 min, as confirmed by HPLC analysis (data not shown). Accordingly, Bauer found that 74% of SA-PN-05 molecules broke down after an irradiation for 20 min (122 J) (Bauer, 2016). The smaller CFU-reductions found for *E. coli* may be due to the general differences in susceptibility of Gram-negative and Gram-positive bacteria toward aPDT (Cieplik et al., 2018a). *S. mutans* and *A. naeslundii* are Gram-positive bacteria which have a cytoplasmic membrane covered by a thick peptidoglycan layer. Both are more vulnerable than *E. coli*, a Gram-negative bacterium, which has a much thinner peptidoglycan layer but, additionally to the cytoplasmic membrane and the peptidoglycan layer, an outer membrane containing lipopolysaccharides (LPS) (Nikaido, 2003; Martínez de Tejada et al., 2012; Spagnul et al., 2015). Due to the differences in cell wall composition, there may also be a difference in accumulation of the PS and therefore an influence on reduction of the bacterial ability to replicate (Misba et al., 2017).

For assessing bacterial membrane damage, flow cytometric analysis was employed with SG I and PI as fluorescent stains. SG I is used as a dye that stains all bacterial cells green whereas PI stains membrane damaged or dead cells with a compromised membranes red (Joux and Lebaron, 2000; Feng et al., 2014). Here, CHX and SA-PN-05 were used as positive controls for membrane damage. Despite the pronounced CFU reductions found for aPDT with SAPYR, there was no increase in PI-positive cells for *A. naeslundii* and *S. mutans*, which is in accordance with a previous study (Cieplik et al., 2018b). However, there was an increase in unstained bacteria in *A. naeslundii* and *S. mutans*. As SG I and PI intercalate with intact DNA and stain bacteria, the increase in unstained bacteria could hint to a breakage of DNA after aPDT treatment which may be caused by formation of ROS. Breaks in single- and double-stranded DNA, and also the disappearance of the plasmid super-coiled fraction upon aPDT treatment were reported in other studies (Fiel et al., 1981; Bertoloni et al., 2000; Hamblin and Hasan, 2004). In contrast to both Gram-positive bacteria, *E. coli* showed an increase of PI-positive cells after treatment with aPDT (36%) although there was a reduction in CFU of only 2 log_10_ steps. As discussed above, this difference could again be explained by the different cell wall compositions of Gram-positive and Gram-negative bacteria with Gram-positive bacteria being potentially less susceptible to membrane damage due to their thicker cell wall (Yin et al., 2015). Furthermore, it is well known that aPDT with cationic PS is able to damage at least the outer membrane of Gram-negative bacteria (Alves et al., 2009; Sperandio et al., 2013). As expected, the positive controls CHX and SA-PN-05 exhibited an increase in the percentage of PI-positive cells for all three tested bacteria.

These findings are in line with the exemplary SEM and TEM visualizations. Analysis of bacterial surface using SEM and TEM showed changes after treatment with aPDT using SAPYR only for *E. coli*. In contrast, no alterations could be observed for *A. naeslundii* and *S. mutans*, which is in accordance with a previous study (Cieplik et al., 2018b). SEM visualization showed that treatment with SA-PN-05 and CHX led to debris and blisters on the bacterial surfaces suggesting bacterial membrane damage, which is also in agreement with data obtained from flow cytometric analysis. TEM analysis revealed the formation of vesicles in *E. coli* upon treatment with CHX. This phenomenon is in accordance with a previous study showing blebbing of the outer membrane resulting in the formation of vesicles in *Pseudomonas stutzeri* upon treatment with CHX (Tattawasart et al., 2000). An explanation may be the formation of so-called bacterial outer membrane vesicles, which are induced by several stress factors like osmotic stress, heat shock, antibiotics, or other antimicrobial agents (Baumgarten et al., 2012; Maredia et al., 2012; Anand and Chaudhuri, 2016).

Besides membrane damage, which could not be accounted for the reduced ability to replicate in *S. mutans* and *A. naeslundii*, a loss in metabolic activity may also be a cause for reduced ability to replicate. We therefore focused on changes in intracellular ATP levels by employing a luminescence assay for measuring ATP concentrations upon treatment with aPDT using SAPYR and with CHX. In all three bacteria, we found a reduction in ATP concentration after irradiation without SAPYR, although bacterial replicability was not affected. Reduction in intracellular ATP concentration after irradiation with blue light was also shown by Abana et al. (2017). In their study, different *E. coli* strains were treated with blue light (455 nm) and there was no reduction in growth, but decreased intracellular ATP despite no increase in extracellular ATP (Abana et al., 2017). In this study, *E. coli* treated with aPDT showed a reduction in ATP concentration of only approximately 50% whereas there was a reduction of 2 to 3 log_10_ steps of CFU. There is a state of bacteria called “viable but non-culturable” (VBNC) which can be caused by stressors like starvation, low temperature, or antibiotics (Biosca et al., 1996). Compared to dead cells, VBNC cells still have an intact cell membrane and an active, but strongly reduced metabolism and still carry out respiration (Del Mar Lleò et al., 2007; Oliver, 2010). Recent studies investigated this VBNC state in *E. coli* (Asakura et al., 2008; Muela et al., 2008; Abana et al., 2017). For instance, Zhao et al. (2016) found that the VBNC state not only leads to loss of bacterial replicability (as measured by culturability) and low metabolic activity but also to downregulated gene and protein expression, DNA replication, cell division, and low ATP levels. In this context, it is noteworthy to mention that the method employed here for detection of ATP may have some limitations because ATP could not be measured directly, but 15 min after treatment. Akhova and Tkachenko (2014) found that there is a temporary rise of ATP within 10 min after treatment with antibiotics accompanied with the induction of antioxidant regulons and inhibition of growth because of an accumulation of ATP due to the inhibition of energy-consuming processes. This could lead to side reactions, for instance to formation of intracellular ROS after treatment (Akhova and Tkachenko, 2014).

Therefore, we measured ROS by means of H_2_DCF-DA, a non-fluorescent dye which is converted into the fluorescent dye DCF in presence of intracellular ROS. Here, we found a concentration-dependent increase in DCF-fluorescence 30 min after aPDT and even a slight increase in the dark controls. These findings are in accordance with other studies, which also showed increase in intracellular ROS after aPDT using H_2_DCF-DA (Misba et al., 2017; Anju et al., 2019; Parasuraman et al., 2019). Normally, there is a balance between generation and elimination of intracellular ROS. If cells are stressed or the electron transport chain is damaged, this balance gets disrupted which in turn causes an increase in generation of intracellular ROS leading to so-called oxidative stress. Oxidative stress may further lead to a subsequent damage of DNA, lipids or proteins (Gonzalez-Flecha and Demple, 1995; Keyer and Imlay, 1996; Liang and Zhou, 2007). Furthermore, oxidative stress is more likely to inhibit bacterial replication than to kill them directly (Imlay, 2015). Consequently, the formation of intracellular ROS 30 min after treatment could explain the loss of bacterial replicability found in this study upon treatment with aPDT.

Likewise, we also found increased formation of ROS 30 min after treatment with CHX in *A. naeslundii and E. coli*. Biswas et al. (2019) reported accordingly that the mode of action of CHX is not just damaging bacterial membrane but also production of ROS. Post-treatment formation of intracellular ROS could be a major factor when it comes to development of tolerance or an altered susceptibility. In this context, sublethal formation of ROS may lead to adaption of bacterial cells to antimicrobial agents due to the activation of general stress response gene elements like the *rpoS* gene-regulon or activation of genes coding for multidrug efflux pumps (Dodd et al., 2007; Fraud and Poole, 2011).

In conclusion, the slight reduction of ATP and the formation of intracellular ROS in biofilms upon treatment with aPDT could present an insight in the mechanism of action. Further studies are necessary to verify this assumption, for instance investigating gene and protein expression upon treatment with aPDT.

## Data Availability Statement

The raw data supporting the conclusions of this article will be made available by the authors, without undue reservation.

## Author Contributions

DM, FC, WB, TM, and K-AH conceived and designed the experiments. CR, SK, HS, and DM performed the experiments. DM, K-AH, FC, and TM analyzed the data. CB and HS performed and analyzed TEM experiments. DM wrote the manuscript with input from all authors. All authors reviewed and approved the final version of the manuscript.

## Conflict of Interest

The authors declare that the research was conducted in the absence of any commercial or financial relationships that could be construed as a potential conflict of interest.
